# ERK Is Involved in the Reorganization of Somatosensory Cortical Maps in Adult Rats Submitted to Hindlimb Unloading

**DOI:** 10.1371/journal.pone.0017564

**Published:** 2011-03-08

**Authors:** Erwan Dupont, Laurence Stevens, Laetitia Cochon, Maurice Falempin, Bruno Bastide, Marie-Hélène Canu

**Affiliations:** 1 Université Lille Nord de France, Lille, France; 2 EA 4488, IFR 114, Université Lille 1, Sciences et Technologies, Villeneuve d’Ascq, France; Tokyo Medical and Dental University, Japan

## Abstract

Sensorimotor restriction by a 14-day period of hindlimb unloading (HU) in the adult rat induces a reorganization of topographic maps and receptive fields. However, the underlying mechanisms are still unclear. Interest was turned towards a possible implication of intracellular MAPK signaling pathway since Extracellular-signal-Regulated Kinase 1/2 (ERK1/2) is known to play a significant role in the control of synaptic plasticity. In order to better understand the mechanisms underlying cortical plasticity in adult rats submitted to a sensorimotor restriction, we analyzed the time-course of ERK1/2 activation by immunoblot and of cortical reorganization by electrophysiological recordings, on rats submitted to hindlimb unloading over four weeks. Immunohistochemistry analysis provided evidence that ERK1/2 phosphorylation was increased in layer III neurons of the somatosensory cortex. This increase was transient, and parallel to the changes in hindpaw cortical map area (layer IV). By contrast, receptive fields were progressively enlarged from 7 to 28 days of hindlimb unloading. To determine whether ERK1/2 was involved in cortical remapping, we administered a specific ERK1/2 inhibitor (PD-98059) through osmotic mini-pump in rats hindlimb unloaded for 14 days. Results demonstrate that focal inhibition of ERK1/2 pathway prevents cortical reorganization, but had no effect on receptive fields. These results suggest that ERK1/2 plays a role in the induction of cortical plasticity during hindlimb unloading.

## Introduction

Cortical maps are highly dynamic structures which can reorganize in response to changes in environmental demands or in sensorimotor experience. For instance, amputation, peripheral nerve lesion or restriction in sensory experience induce remodeling of the topological cortical maps [1]. Such a remodeling is also described in the somatosensory cortex of adult rats submitted to hindlimb unloading (HU) [2], [3], a situation commonly used in rats to mimic the effects of confinement to bed in patients, or even space-flight.

During HU, the contact of the plantar sole of hindlimb with the ground is prevented and thus the tactile information from the paw and the proprioceptive input from the limb are dramatically reduced [4]–[6]. As previously described by our group, the sensorimotor restriction obtained by a 14-day period of HU induces a reorganization of cortical maps, characterized by a shrinkage of the foot representation area and an enlargement of cutaneous receptive fields (RF) [2], [3].

Although the molecular events involved in this plasticity are still obscure, it has been shown that the expression of neurotrophins was increased in HU rats [7]. The transduction of neurotrophin extracellular signal from surface receptors to regulatory targets within the cytoplasm and the nucleus of the cell is mediated by Mitogen-Activated Protein Kinases (MAPKs) [8], [9]. Among MAPKs, extracellular-signal-regulated kinase 1/2 (ERK1/2) signaling pathway is described as a key regulator of neuronal function. ERK1/2 plays a critical role in the control of synaptic plasticity in the developmental and mature brains [10], [11]. In particular, the role of ERK1/2 in long-term potentiation (LTP) is now clearly established [11]–[14]. However, we have no data about the potential implication of ERK1/2 in the remodeling of cortical somatotopic maps. According to the increase in neurotrophin levels during HU and to their potential role in the activation of the MAPK cascades, we hypothesize that MAPKs activation could be modified in the somatosensory cortex and play a significant role in the cortical plasticity in adult mammals. Thus, the objectives of the present study were threefold. Our first objective was to establish a time-course of cortical reorganization of adult rats submitted to 7 to 28 days of HU. In fact, although previous papers have described the changes in cortical somatotopic representation of hindlimbs after a 14-day period of HU, the time-course of changes is unknown. The second objective was to perform in parallel a time-course of the MAPK activation. The third objective was to determine whether focal inhibition of ERK1/2 pathway with PD98059 prevented cortical reorganization. PD98059 is a highly selective inhibitor of MAP kinase kinase activation, resulting in decreased phosphorylation of ERK1 and ERK2 [15], Our main conclusion is that molecular mechanisms of cortical map plasticity involve ERK1/2 activation.

## Methods

### Ethics statement

All procedures described below were approved by both the Agricultural and Forest Ministry and the National Education Ministry (veterinary service of health and animal protection, authorization 59-00999). All efforts were made to minimize suffering.

### Animals and treatment

Adult male Wistar rats (280–320 g) were divided into four groups: C (control), HU7, HU14 and HU28 (Hindlimb Unloading for 7, 14 and 28 days, respectively). The animals were housed under temperature and light controlled conditions (23°C, 12-h light/12-h dark cycle). Hindlimb unloading was performed using the tail suspension model [16]. This situation prevented the contact of the hindlimbs with the ground, whereas the rats were allowed to walk freely on their forelimbs and they had *ad libitum* access to food and water.

### Chronic infusion of ERK1/2 inhibitor

Some animals of the C and HU14 groups received a unilateral chronic infusion in the right cortex of PD-98059 (50 µM, Calbiochem) (C-PD98059 and HU14-PD98059 subgroups) or vehicle (1% dimethyl sulfoxide in artificial cerebrospinal fluid) (C-Vehicle, and HU14-Vehicle subgroups). Delivery was achieved via a cannula attached with a flexible catheter to an osmotic minipump (model 2002, Alzet). Implantation was made under sodium pentobarbital anesthesia (60 mg/kg, i.p.) as previously described [17]. Briefly, the cannula was inserted in the cortex at coordinates anterior −3.5 and lateral 3 with Bregma as reference point, at a depth of 800 µm, and fixed to the skull with dental cement. The pump was placed subcutaneously on the back of the rat. The skin was sutured, and an antiseptic (Betadine®) was applied on incision area. Rats were allowed to recover for one day before suspension.

### *In vivo* electrophysiology

Electrophysiological recordings were performed on 56 rats (C: *n* = 11; C-Vehicle: *n* = 3; C-PD98059: *n* = 4; HU7: *n* = 5; HU14: *n* = 12; HU14-Vehicle: *n* = 7; HU14-PD98059: *n* = 7; HU28: *n* = 7) to determine the extent of the hindpaw representation on the somatosensory cortex. The detailed procedure is described elsewhere [17]. The rats were anaesthetized with sodium pentobarbital (60 mg/kg, i.p.). Multiunit recording was performed with tungsten microelectrodes (5 MΩ at 1 kHz) in layer IV (depth: 700–1200 µm). The activity was amplified (×10000), filtered (bandwidth 0.3–10 kHz, model 1800, AM-Systems), visualized on an oscilloscope, and delivered to an audio monitor. For each penetration site, the skin of the foot was hit with force-calibrated Semmes-Weinstein monofilaments (Stoelting Co.). Cutaneous RF were defined as regions where low threshold tactile stimulation (<400 mg) systematically enhanced the neural activity. The cortical representation of the hindpaw was reconstructed by drawing boundaries to enclose the sites responding to hindpaw stimulation. The hindpaw cortical area and the surface of the cutaneous RF were determined by using Photoshop software.

### Western blot analyses

Western blot analyses were performed on 62 rats (C: *n* = 10; C-Vehicle: *n* = 4; C-PD98059: *n* = 4; HU7: *n* = 8; HU14: *n* = 9; HU14-Vehicle: *n* = 8; HU14-PD98059: *n* = 8; HU28: *n* = 11) to quantify the level of total and phosphorylated forms of ERK1/2.

#### Cortex sampling

The total duration of the removal did not exceed 4 min to prevent potential changes in ERK activation due to ischemia. In addition, in order to avoid a possible effect of anesthesia on ERK1/2 phosphorylation states, rats were sacrificed by decapitation. The head was immediately surrounded with ice and placed in a stereotaxic frame. A craniotomy was performed to expose the cerebral cortex. The dura-mater was resected. A plastic cylinder (1 mm inner diameter) mounted on a syringe was used to remove by aspiration a column of hindpaw somatosensory cortex at the stereotaxic coordinates anterior −1 and lateral 3 with Bregma as bone reference point. These coordinates correspond to the functional center of the hindpaw cortical map in C and HU14 groups as determined in a prior study [18]. A column of visual cortex was also removed at the stereotaxic coordinates anterior −5 and lateral 3 to serve as internal control. The somatosensory and visual cortices were immediately frozen in liquid nitrogen and stored at −80°C for later use.

#### Preparation of tissue extracts

The frozen brain samples were dropped in 200 µl of lysis buffer containing 50 mM Tris-HCl, pH 7.4, 0.1% Triton X-100, 4 mM EGTA, 10 mM EDTA, 100 mM β-glycerophosphate, 5 mM sodium orthovanadate, 15 mM tetrasodium pyrophosphate, 25 mM sodium fluoride, 15 µg/µl leupeptin, 15 µg/µl aprotinin, 15 µg/µl pepstatin. The samples were homogenized with an ultrasonic cell disrupter and were centrifuged at 13000× *g* for 10 min at 4°C. The protein concentration was determined by a Bradford assay.

#### Antibodies

Primary antibodies against phosphorylated (P-ERK1/2) and total forms of ERK1/2 were purchased from Cell Signaling Technology (#9101 and #9102 respectively). P-ERK1/2 antibody detects endogenous levels of ERK1 and ERK2 when phosphorylated either individually or dually at Thr^202^ and Tyr^204^ residues. The antibody does not cross-react with non-phosphorylated ERK1/2. The primary antibody against a housekeeping protein (α-tubulin) was purchased from Sigma (T6199). HRP-conjugated anti-rabbit or anti-mouse secondary antibodies were purchased from Cell Signaling Technology (#7074 and #7076 respectively).

#### Immunoblotting

The samples (20 µg per lane) were then diluted in SDS-PAGE sample buffer (50 mM Tris-HCl, pH 6.8, 2% SDS, 10% glycerol, 5% β-mercaptoethanol and 0.1% bromophenol blue), heated 3 min at 95°C and resolved on 10% SDS polyacrylamide gels. The proteins were transferred to a 0.2 µm nitrocellulose membrane (Biorad). The membrane was blocked with 5% non-fat dry milk in Tris-buffered saline (TBS) for 2 h at room temperature.

The same membrane was processed in three sequential steps: incubation with (i) antibody against phosphorylated form of ERK1/2; (ii) antibody against the total form of ERK1/2; and (iii) antibody against α-tubulin. Blots were firstly incubated overnight at 4°C with anti-P-ERK1/2 antibody (1∶1000). For detection, the membranes were incubated with HRP-conjugated anti-rabbit secondary antibody (1∶2000) for 1 h at room temperature. Immunoreactivity was detected using enhanced chemiluminescence (PerkinElmer). Blots were stripped with Western Re-probe buffer (Agro-Bio) and then re-probed with anti-ERK1/2 antibody (1∶2000). Blots were incubated with HRP-conjugated anti-rabbit secondary antibody (1∶2000) and visualized as described above. Finally, following a second stripping step, blots were incubated overnight at 4°C with anti-α-tubulin primary antibody (1∶10000) and thereafter with HRP-conjugated anti-mouse secondary antibody (1∶5000). The signals were revealed as above to check that equal amounts of proteins were loaded on the gel.

#### Densitometric analysis

All blots were scanned and densitometric analysis was conducted using GS-800 Imaging densitometer and QuantityOne Software (Biorad). Both ERK1/2 and P-ERK1/2 were normalized to α-tubulin, which was used as an internal control. For the different HU periods, ERK1/2 expression was compared to its basal expression in the C group as previously described [19].

### Immunohistochemistry

In order to localize P-ERK1/2-immunoresponsive cells within cortical layers, immunostaining was performed on 4 rats in C and HU14 groups (*n* = 2 for each group). The rats were anesthetized with sodium pentobarbital (as described above) and transcardiac perfusion was performed with ice-cold fixative solution containing 4% paraformaldehyde in 0.1 M Na_2_HPO_4_/NaH_2_PO_4_ buffer, pH 7.5. Brains were quickly removed and postfixed overnight in the same solution at 4°C. Coronal sections from stereotaxic coordinates A −0.5 to A −1.5 (50 µm thick) were cut with a vibratome (WPI) in TBS (25 mM Tris-Cl, 150 mM NaCl, pH 7.4). For the detection of phosphorylated proteins, 50 mM NaF was included in all buffers and incubation solutions. Between each treatment, sections were washed a minimum of 4× (5 min per wash) in TBS. Initially, free-floating sections were incubated with 1% H_2_O_2_ in TBS for 30 min to inactive endogenous peroxidase activity. Then, tissues were saturated for 1 h with TBS containing 3% bovine serum albumin and 0.2% Triton X-100. The sections were incubated overnight at 4°C with mild agitation in TBS containing anti-P-ERK1/2 (1∶2000). The next day, the reaction was completed with incubation in biotinylated anti-rabbit secondary antibody (1∶2000; Vector Laboratories) for 2 h at room temperature, and then in an avidin-biotin enzyme complex (Vectastain ABC kit; Vector Laboratories) for 2 h at room temperature. Nickel-intensified diaminobenzidine was used to visualize the signal. After processing, the tissue sections were mounted onto gelatin-coated slides and dehydrated through alcohol to toluene and coversliped using Eukitt (Chem-Lab) for light microscopic examination.

We defined two regions of interest on the same sections: the hindpaw somatosensory cortex (stereotaxic coordinates lateral 2.5 to 3.5) and the precentral medial area, used as reference (lateral 0.4 to 1). The density of P-ERK1/2-immunopositive cells was measured by counting the cell number in these two regions, principally on layers II/III. We considered all detectably labeled cells as P-ERK1/2-immunoresponsive cells. In all experiments, the number of immunopositive cells was counted in a blind manner in relation to experimental conditions.

### Statistical analyses

Results are presented as mean ± SEM. Normality was tested by Kolmogorov-Smirnov test. For the time-course of cortical area changes, one-way analysis of variance (ANOVA) was used. Receptive fields extent was compared with the non-parametric Kruskall-Wallis test followed by Dunn’s post-hoc test. Multi-group results were compared by a two-way ANOVA. Independent variables were *Group* (CON *vs.* HU) and *Cortex* (somatosensory *vs.* visual) for the time-course of ERK1/2 activation; *Group* (CON *vs.* HU) and *Cortex* (somatosensory *vs.* precentral medial area) for immunohistochemistry experiment; *Group* (CON *vs.* HU) and *Substance* (Vehicle *vs.* PD98059) for the effects of PD98059 infusion on cortical area, on RF extent, and on ERK1/2 activation. The Tukey test was used for post-hoc comparison. A *P*-value of less than 0.05 was chosen as the significance level for all statistical analyses.

## Results

### Time-course of cortical reorganization

**Figure 1:**
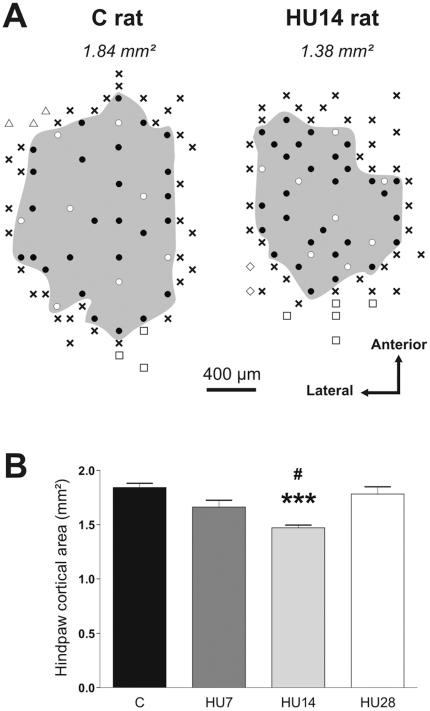
Hindlimb unloading induces a reorganization of somatosensory hindpaw cortical area. (A) Representation of the hindpaw area in the somatosensory cortex for one C and one HU14 rats. • and ○ indicate locations of penetrations where cortical neurons had hindpaw cutaneous receptive fields. ○ corresponds to the recording sites of the 8 cutaneous receptive fields presented in figure 2. **×** indicates penetrations where neurons were not responsive to cutaneous stimulation. The *grey area* shows the cortical representation of the hindpaw (values expressed in mm^2^ on top). The penetration sites for adjacent body regions are represented by ▵ (forelimb), □ (hindlimb) and ⋄ (trunk). (B). Mean value of the hindpaw cortical area for C and HU-x groups. 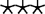 indicates a significant difference between C and HU14 groups (*P*<0.001), and # between HU14 and HU28 groups (*P*<0.05).

Figure 1 presents typical examples of the hindpaw cortical area for C and HU14 rats (figure 1A), and the mean size of this area for all the groups (in mm^2^) (figure 1B). The one-way ANOVA showed a difference between the groups (*P*<0.001). In the C rats, the hindpaw representation occupied a mean area of 1.84±0.04 mm^2^. We observed a progressive decrease at 7 and 14 days of HU (respect. −9.8%, *ns*; −15.4%, *P*<0.001). For the HU28 rats, the hindpaw cortical area was similar to that of C rats (−3.3%, *ns*).

**Figure 2:**
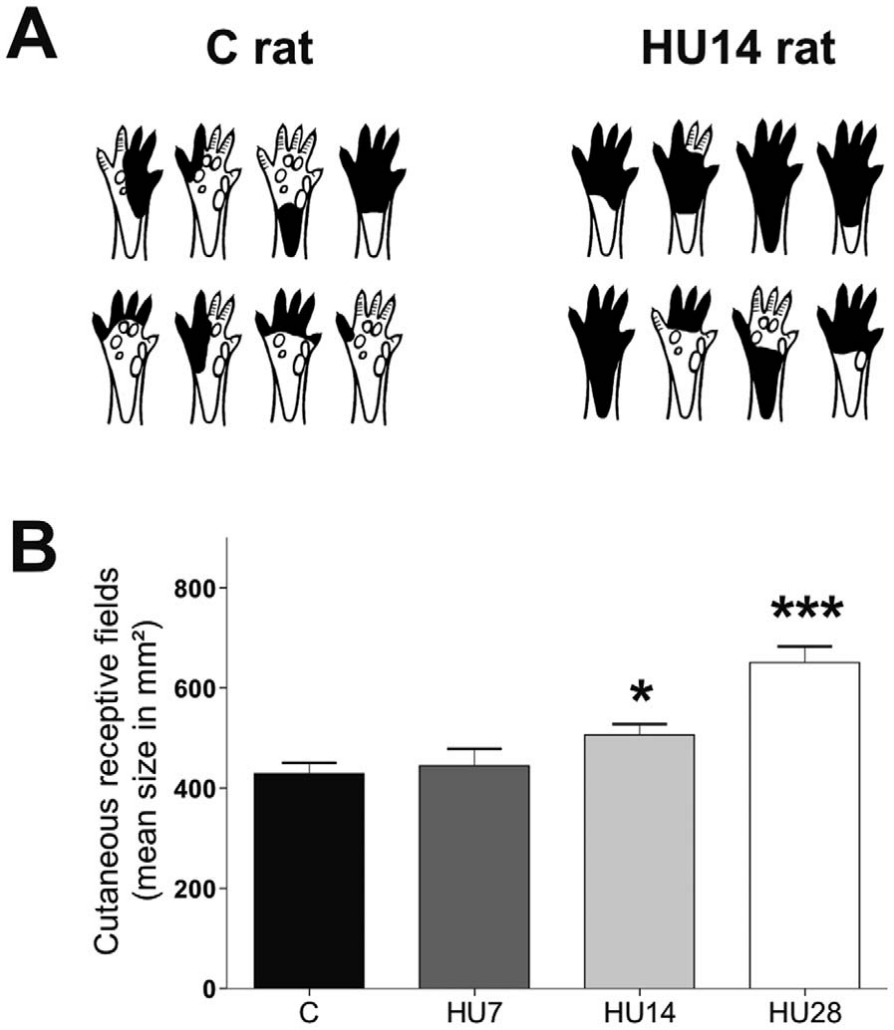
Hindlimb unloading induces an extension of the cutaneous RF size. (A) Illustration of a sample of cutaneous RF for one C and one HU14 rats, obtained at the penetration sites presented in figure 1 (*white dots*). Only the ventral surface [42] of the hindpaw is presented. (B) Cutaneous RF mean size (mm^2^) for C and HU-x groups. 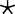 and 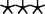 indicate a significant difference from C group (*P*<0.05 and *P*<0.001).

Figure 2 presents examples of typical cutaneous RF for C and HU14 rats (figure 2A), and the mean size of these fields (in mm^2^) for all the groups (figure 2B). The Kruskall-Wallis test showed a difference between the groups (*p*<0.001). In the C rats, the RF mean size was 428±22 mm^2^. We showed a progressive enlargement of the RF across the different periods of HU (HU7: +4%, *ns*; HU14: +19%, *p*<0.05; HU28: +52%, *p*<0.001).

### HU induces a transient increase of ERK1/2 activation in the somatosensory cortex

**Figure 3:**
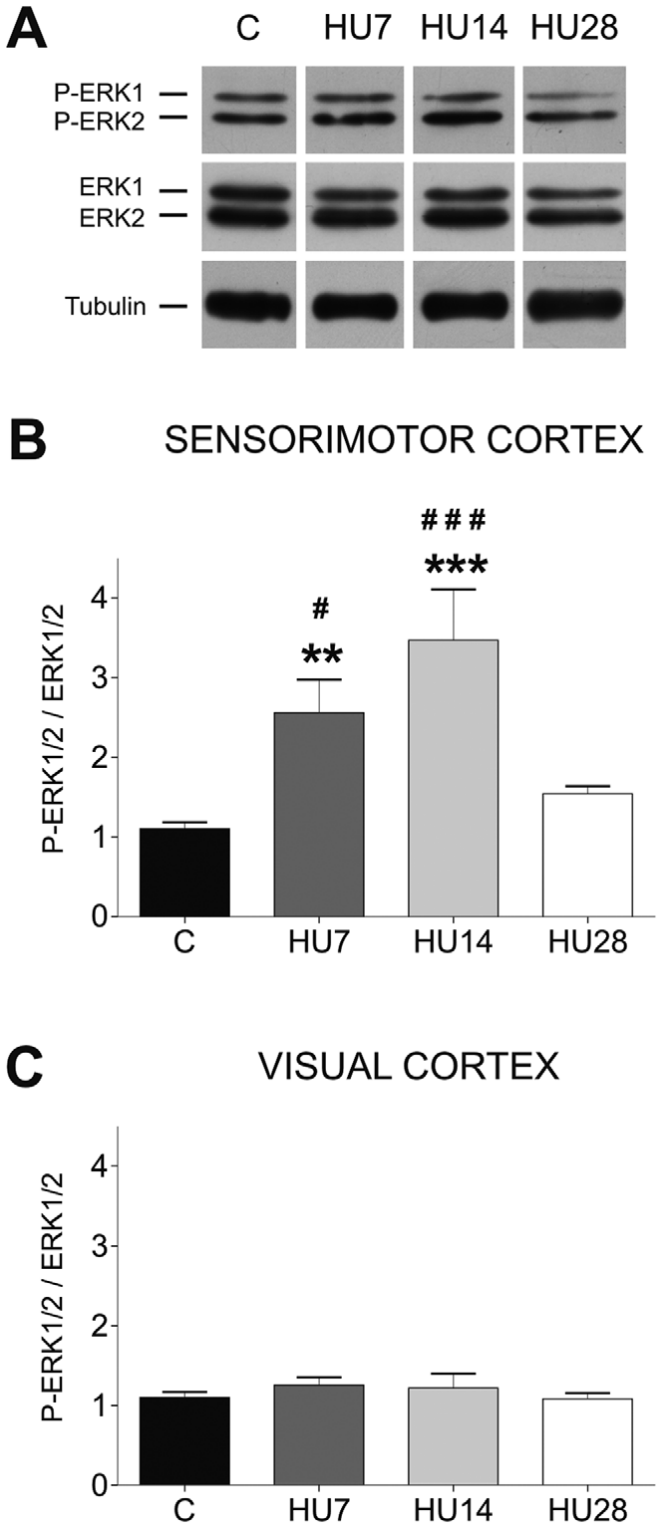
Hindlimb unloading transitory upregulates ERK1/2 phosphorylation in the somatosensory cortex. (A) Representative western blot showing P-ERK1/2 and total ERK1/2 immunoreactivity in the somatosensory cortex along the periods of HU. Time-course of ERK1/2 activation (ratio between P-ERK1/2 and ERK1/2) in the somatosensory cortex (B) and the visual cortex (C) after different periods of HU. 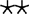 *P*<0.01) and 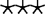 (*P*<0.001) indicate a significant difference from C group. # (*P*<0.05) and ### (*P*<0.001) indicate a significant difference from HU14 group.

In the somatosensory cortex, we observed a significant increase in the ERK1/2 activation (figure 3A and B) after hindlimb unloading. The two-way ANOVA reveals that there was a *Group* effect (*F* = 11.25, *p*<0.001), a *Cortex* effect (*F* = 37.56, *p*<0.001), and a *Group* x *Cortex* interaction (*F* = 8.44, *p*<0.001). ERK1/2 phosphorylation level was expressed as the ratio between P-ERK1/2 and ERK1/2. It was significantly increased for HU7 (+132%, *p*<0.01) and HU14 (+215%, *p*<0.001) groups in comparison to control rats, but returned toward basal level for HU28 rats (+40%, *ns*). These changes in P-ERK1/2 immunoreactivity were not due to alterations in total ERK1/2 levels since ratio between total ERK1/2 and α-tubulin did not change between all the groups (figure 3A). In the visual cortex (figure 3C), the activation and expression of ERK1/2 were similar to values observed in the somatosensory cortex in C rats, and remained unchanged during HU. By consequence, the changes described above are specific to somatosensory cortex, and are not a generalized activation of the ERK1/2 signaling pathway by HU in the whole cortex.

**Figure 4:**
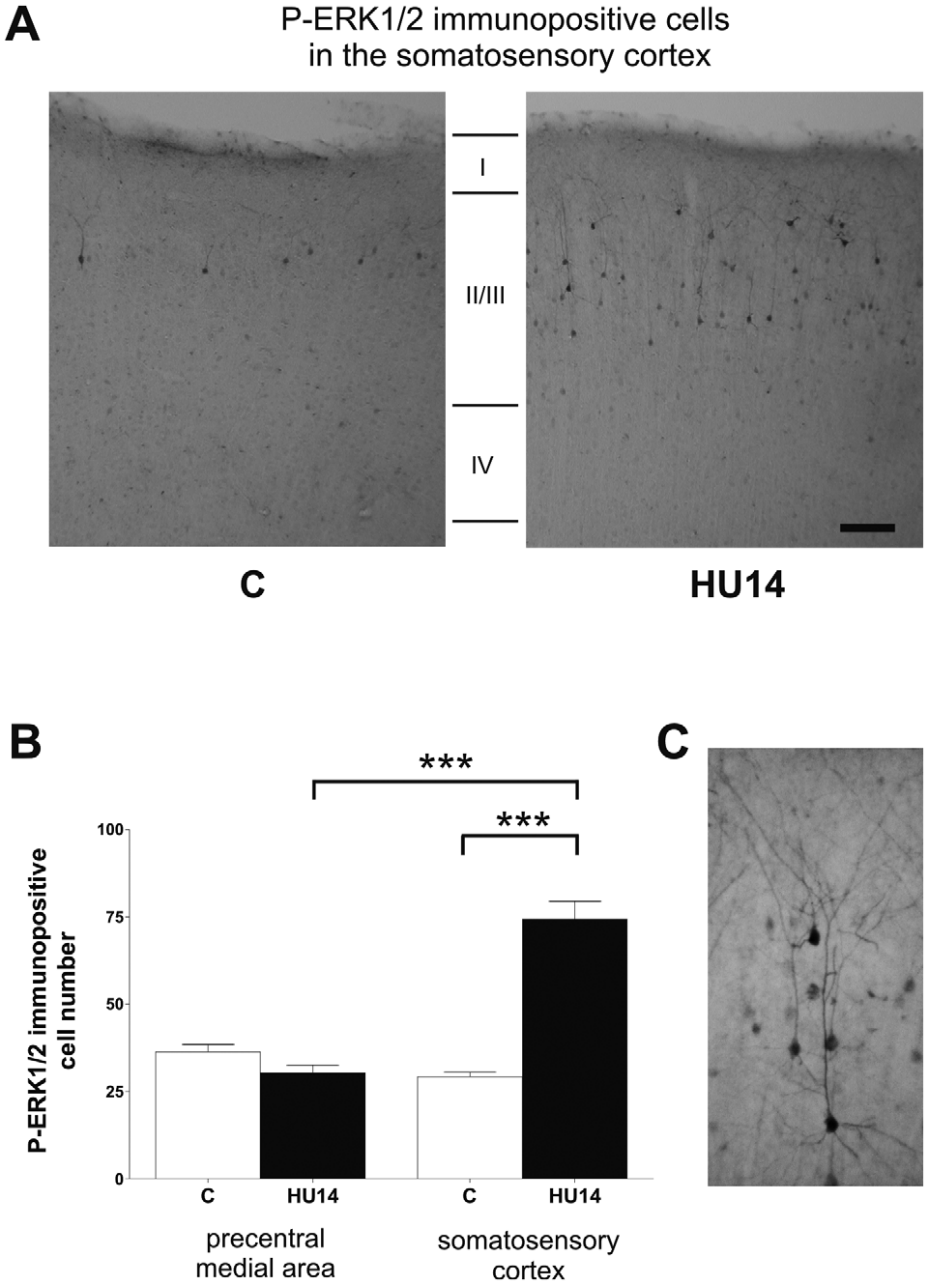
Hindlimb unloading increases ERK1/2 phosphorylation in layer II/III of the somatosensory cortex. (A) Representative photographs showing the spatial localization of P-ERK1/2 in the somatosensory cortex of C and HU14 rats. The coronal section was performed at stereotaxic coordinates A −1 from bregma. (B) Expression of the P-ERK1/2 immunopositive cell number in precentral medial area (used as reference) and the somatosensory cortex of C and HU14 rats. (C) High-magnification photograph of P-ERK1/2 immunopositive neurons showing staining in cell body and dendrites. 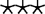 indicates significant differences between cortical regions and between C and HU14 groups (*P*<0.001).

In order to determine the precise localization of cells expressing P-ERK1/2 within the cortical layers, we performed an immunohistochemical study in C and HU14 rats. The analysis revealed that in C rats, the stained cells were mainly observed in layers II/III and hardly found in layer VI (figure 4A). We were not able to detect staining in layers IV and V. High magnification photographs revealed that P-ERK1/2 was detected in both dendrites and cell body (figure 4C). In C rats, cells immunopositive for P-ERK1/2 were distributed at a similar density in the somatosensory cortex and the precentral medial area (figure 4B). In HU14 rats, the proportion of these cells strongly increased in the somatosensory cortex (+154%, *p*<0.001), whereas no change was noticed in the precentral medial area (−19%, *ns*). The two-way ANOVA indicates that there was a *Group* effect (*F* = 40.01, *p*<0.001), a *Cortex* effect (*F* = 35.78, *p*<0.001), and a *Group* x *Cortex* interaction (*F* = 68.47, *p*<0.001).

### Focal inhibition of ERK1/2 prevents reorganization of cortical maps

**Figure 5:**
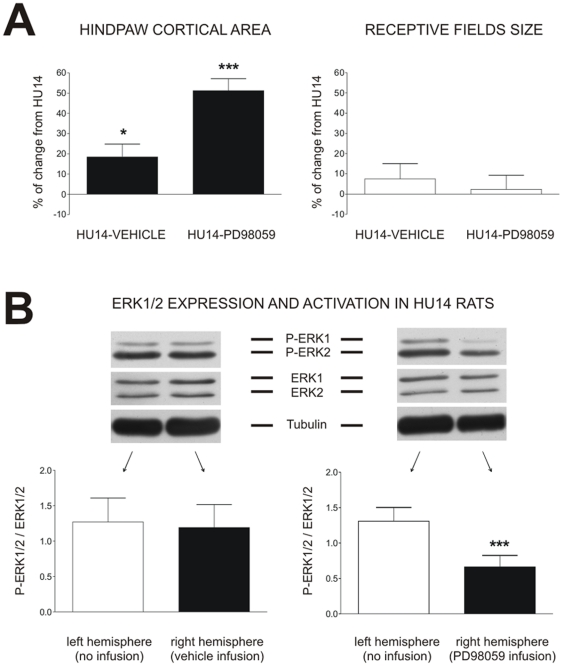
ERK1/2 inhibition prevents cortical map plasticity induced by hindlimb unloading. (A) Effect of vehicle or PD98059 ERK1/2 inhibitor chronic infusion in the right somatosensory cortex on the hindpaw cortical area and the receptive fields size. Data are expressed as percentage of change from HU14 group. 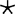 (*P*<0.05) and 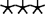 (*P*<0.001) indicate a significant difference from HU14 group. (B) Effect of vehicle or PD98059 ERK1/2 inhibitor chronic infusion in the right somatosensory cortex on ERK1/2 expression and activation. Representative western blots of P-ERK1/2, total ERK1/2 and α-tubulin expression in the left and right somatosensory cortices, and histograms showing ERK1/2 activation (ratio between P-ERK1/2 and ERK1/2). 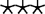 (*P*<0.001) indicates a significant difference between left and right somatosensory cortices.

Since P-ERK1/2 level varied in parallel with the area of hindlimb representation, the next step was to determine whether a focal inhibition of ERK1/2 pathway by means of its specific inhibitor PD-98059 could prevent cortical remapping. In C rats, vehicle or PD98059 infusion during a 14-day period had no effect (−0.6% and +0.4% respectively, *ns*) on the extent of hindpaw representation on the cortex. In HU14 rats, PD98059 infusion dramatically increased the hindpaw cortical area (+51.2%, *p*<0.001) (Figure 5A). It should be noted that vehicle slightly affected the cortical map (+18.4%, *p*<0.05). Values in HU14-PD98059 rats were higher to that observed in C animals (+27.9%, *p*<0.001). The two-way ANOVA reveals a *Substance* effect (*F* = 13.56, *p*<0.001) and a *Group* x *Substance* interaction (*F* = 13.09, *p*<0.001).

ERK inhibitor did not affect the extent of receptive fields (Figure 5A). The two-way ANOVA indicates a *Group* effect (*F* = 7.81, *p*<0.01), but no *Substance* effect (*F* = 2.22, *ns*) and no *Group* x *Substance* interaction (*F* = 1.94, *ns*).

To evaluate whether chronic infusion of PD98059 actually inhibited ERK1/2 activation, we measured the ratio P-ERK1/2/ERK1/2 in cortical samples taken in the left and right hindpaw cortical areas (figure 5B). After injection of vehicle, the ratio P-ERK1/2/ERK1/2 was reduced in both hemispheres with respect to HU14 values, revealing a sham effect. However, after infusion of PD98059, the activation level of ERK1/2 in the right cortex was dramatically decreased with respect to HU (−84%, *p*<0.001), and HU-Vehicle (*P*<0.05) groups. This effect of pump implantation on ERK1/2 activation could account for the slight changes in cortical map area in the HU14-Vehicle group: the lower the activation level of ERK is, the larger the map is.

## Discussion

The main objective of the present study was to determine whether ERK1/2 signaling pathway was involved in the reorganization of cortical maps in HU rats. We found that ERK1/2 phosphorylation increases in layer III neurons during HU. This increase is transient, and is parallel to the changes in representation of hindpaw in the layer IV of the somatosensory cortex. Finally, we demonstrated that inhibition of ERK1/2 signaling pathway prevents cortical remapping. Taken together, this study suggests that this MAPK cascade member plays a role in the cortical plasticity induced by a decrease in sensorimotor activity. To our knowledge, although the role of ERK1/2 in LTP is largely documented, this is the first evidence for an implication of ERK1/2 activation in remapping of somatosensory cortex in adult rats.

### 1-Hindlimb unloading increases ERK1/2 phosphorylation

HU can have an action on signaling pathways through several neuronal events. Binding of trophic factors on tyrosine kinase receptors might activate ERK1/2 [20]. Previous data demonstrated that mRNA and protein levels of NGF were increased after 14 days of HU [7]. G-protein coupled cholinergic receptors are also strong activators of ERK1/2. In particular, activation of muscarinic receptors in rat hippocampal slices [21] and in primary cortical cultures [22] induces prolonged activation of ERK1/2. Cholinergic mechanisms are known to be involved in reorganization of somatotopic cortical maps [23], and blockade of muscarinic receptors prevents cortical remapping [17]. However, in somatosensory cortex, muscarinic receptors are mainly located in layer V, and to a lesser extend in supragranular layers [24] whereas in our study, cells stained for P-ERK1/2 were encountered almost exclusively in layer II/III. In consequence, it is unlikely that cholinergic neurotransmission plays a direct role in the activation of MAPK during HU. Glutamate also increases MAPK phosphorylation via NMDA and AMPA receptors [8], [10]. However, we have shown that the level of glutamate was unchanged in the somatosensory cortex of rats submitted to 14 days of HU [25]. On the other hand, GABA inhibition is reduced during HU [25]. This lower inhibition, which concerns mainly layer V cells [26], might activate neurons of supragranular layers, through broad excitatory projection from layer V to layers II/III [27].

### 2-ERK1/2 is activated in supragranular layers whereas map reorganization is observed within granular layer

Yet, how an increase in ERK1/2 phosphorylation in layer III might affect cortical maps in layer IV? Pyramidal neurons of layer III project onto inhibitory interneurons of layer IV [27]. Thus, layer IV interneurons can integrate direct thalamocortical input with the inputs they receive from layer III neurons, which are P-ERK1/2 immunopositive cells. Supragranular layers play a role in topographic organization and contribute to the plasticity of underlying layers [28], [29]. Besides, phosphorylation of ERK1/2 seems to be a critical event in the induction, but not in the expression, of LTP [13]. Taken together, these data suggest that the activity of layer IV neurons (and hence the organization of somatotopic maps within this layer) are controlled by supragranular layers.

### 3-ERK1/2 staining is observed at the cytoplasmic level

The strong staining of P-ERK1/2 in dendrites and cell body is consistent with a direct action of MAPK at the cytoplasmic level. ERK1/2 acts at synaptic level where it regulates neurotransmitter release through modulation of the activity of synaptic proteins or ionic channels, and changes in dendritic spine structure [30]–[33]. In the same way, using electron microscopy, it has been demonstrated that visual cortical plasticity depends on ERK1/2 activation in the cytoplasmic compartment [30]. Whether plastic mechanisms in HU rats requires translocation of ERK1/2 to the nucleus remains to be determined. When translocated to the nucleus, P-ERK1/2 activates regulators of gene expression, such as transcription factors crucial for plasticity (CREB, c-fos, c-jun, elk1…) [10], [34]. In a previous study, we did not detect any variation in the number of Fos-immunoreactive neurons in basal conditions after 14 days of HU [35], but we cannot exclude the possibility that MAPK target another transcription factor. Another argument in favor of a transcription independent mechanism is that the action of kinases is transient and lasts about 2 h; after that delay, translocation is necessary to activate new protein synthesis, and for establishing long-lasting changes. However, changes in cortical maps in HU rats are very labile, and a restoration of a normal representation of hindpaw occurred within a few hours (3 h) of return to quadrupedal position [3]. The lack of long-term changes in cortical maps could be explained by an absence of nuclear action of MAPK.

### 4-Changes in cortical reorganization and in ERK1/2 phosphorylation are transient

The shrinkage of somatotopic cortical representation of the hindpaw after 14 days of HU has already been reported [2], [3]. We provide here additional information with the determination of hindpaw cortical area and RF after 28 days of HU. Surprisingly, the hindpaw cortical area expanded between day-14 and day-28, where it reached similar values to that observed in control rat, and in the same way, the phosphorylation level of ERK1/2 returned to control values at HU28. Thus, map reorganization and ERK1/2 activation are both activity-dependent phenomena, but in opposite ways: a reduced sensorimotor activity increases ERK1/2 activation and decreases the cortical map.

Such a transient change in cortical map has already been reported in the motor cortex [36]. Indeed, cortical maps underwent changes during motor learning, and then returned to baseline while the motor skill was retained. The authors concluded that successful acquisition but not storage of a motor skill depends on cortical map changes. We can suggest that this is also applicable to somatosensory cortex. The recording of neurogram might bring another explanation. The activity recorded at the fifth lumbar root of the spinal cord (which innervates the hindlimb) is decreased during the first days of HU, and recovers normal levels within ∼10 days [4], [37]. It is likely that the thalamocortical input is dependent on the activity of peripheral sensory nerves and varies accordingly. Thus, since map reorganization and ERK1/2 activation are activity-dependent phenomena (see above), a return to a normal amount of activity within the cortex might restore a cortical foot area and a level of P-ERK1/2 similar to control values.

This brings us to the question of whether ERK1/2 phosphorylation and cortical remapping are independent processes or if there is a cause and effect relationship between these two phenomena. The administration of ERK1/2 inhibitor PD98059 clearly demonstrates that cortical plasticity requires the activation of ERK1/2 signaling pathway.

### 5-Cortical map changes and RF enlargement are independent processes

At contrast to what was observed for cortical map, the enlargement of RF was more accentuated at HU28 that at HU14, indicating a different time-course for RF shaping and topographic mapping. In addition, PD98059 affected cortical maps but had no effect on RF. An evolution of RF and cortical area on different time scales has already been reported [3], [38]. This finding indicates that mechanisms controlling the two phenomena are probably different. It suggests that the topographic expansion/restriction of cortical maps is closely related to the global level of cortical activity, whereas RF tuning is more dependent of degree of local synchrony of concurrent sensory inputs delivered to the cortex [1]. This hypothesis is supported by previous observations on forelimbs [39]. During HU, forelimbs are essentially engaged in a postural activity rather than in manipulation or grooming; in consequence, the pattern of activity is changed from a phasic to a tonic pattern, without changes in the total activity of the nerve. As a matter of fact, the forelimb cortical area was unchanged whereas RF were enlarged.

To conclude, the present study provides evidence for a role of ERK1/2 in changes in topographic cortical maps in response to a sensorimotor restriction, whereas ERK1/2 is not implicated in RF tuning. These results might help to develop pharmacological agents aimed to favor cortical reorganization after a peripheral lesion or a change in activity, or on the contrary to relieve the effects of disuse on the central nervous system, in particular to attenuate adverse perceptual phenomena, such as “phantom-limb pain” [40] or tinnitus [41].
